# Tetrameric structure of SUR2B revealed by electron microscopy of oriented single particles

**DOI:** 10.1111/febs.12097

**Published:** 2013-01-27

**Authors:** Constantina Fotinou, Jussi Aittoniemi, Heidi Wet, Ange Polidori, Bernard Pucci, Mark S P Sansom, Catherine Vénien-Bryan, Frances M Ashcroft

**Affiliations:** 1 Department of Physiology, Henry Wellcome Centre for Gene Function, University of OxfordUK; 2 Department of Biochemistry, University of OxfordUK; 3 Laboratoire de Chimie Bioorganique Etudes Systèmes Moléculaires Vectoriels, Universitéd'Avignon et des Pays du Vaucluse8400, Avignon, France; 4 IMPMC, UMR 7590 CNRS-Universite P. et M. Curie, 4, Place Jussieu, F-75005Paris, France

**Keywords:** ABC transporter, fluorinated lipid monolayer, ion channel, K_ATP_ channel, sulfonylurea receptor

## Abstract

The ATP-sensitive potassium (K_ATP_) channel is a hetero-octameric complex that links cell metabolism to membrane electrical activity in many cells, thereby controlling physiological functions such as insulin release, muscle contraction and neuronal activity. It consists of four pore-forming Kir6.2 and four regulatory sulfonylurea receptor (SUR) subunits. SUR2B serves as the regulatory subunit in smooth muscle and some neurones. An integrative approach, combining electron microscopy and homology modelling, has been used to obtain information on the structure of this large (megadalton) membrane protein complex. Single-particle electron microscopy of purified SUR2B tethered to a lipid monolayer revealed that it assembles as a tetramer of four SUR2B subunits surrounding a central hole. In the absence of an X-ray structure, a homology model for SUR2B based on the X-ray structure of the related ABC transporter Sav1866 was used to fit the experimental images. The model indicates that the central hole can readily accommodate the transmembrane domains of the Kir tetramer, suggests a location for the first transmembrane domains of SUR2B (which are absent in Sav1866) and suggests the relative orientation of the SUR and Kir6.2 subunits.

**Structured digital abstract:**

- SUR2B and SUR2B bind by electron microscopy (View interaction)
- SUR2B and SUR2B bind by molecular sieving (View interaction)

## Introduction

ATP-sensitive potassium (K_ATP_) channels couple changes in cell metabolism to electrical activity of the plasma membrane [1–3]. Metabolic inhibition leads to K_ATP_ channel opening and suppression of electrical activity, whereas enhanced cellular metabolism promotes K_ATP_ channel closure and stimulates electrical activity and cellular responses. K_ATP_ channels thereby control insulin secretion from pancreatic beta-cells, protect against cardiac stress and brain seizures, mediate ischaemic preconditioning in heart and brain, and set the tone of vascular smooth muscle.

K_ATP_ channels are of major medical importance. Mutations in K_ATP_ channel genes result in a range of diseases including neonatal diabetes, hyperinsulinism and cardiomyopathy [1,4,5]. K_ATP_ channels also serve as the target for the sulfonylurea drugs, which are used routinely to treat type 2 diabetes [6]. These drugs bypass cell metabolism and promote insulin secretion by binding directly to the channel and inhibiting K_ATP_ channel activity. Conversely, binding of K-channel openers enhances channel opening [7].

**Fig. 1:**
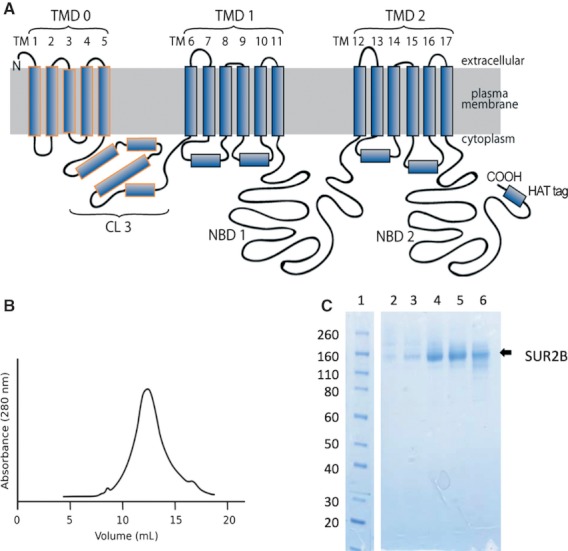
Purification of tetrameric SUR2B. (A) Topology map of SUR, showing the three sets of transmembrane domains (TMD0, TMD1 and TMD2) and the nucleotide-binding domains (NBD1, NBD2). (B) Elution profile of rat SUR2B by size-exclusion chromatography using a Superose 6 column after affinity purification. (C) Coomassie-stained SDS/PAGE (4–12% Bis-Tris) denaturing gels of SUR2B: lane 1, molecular weight markers (kDa); lanes 2–6, main peak fractions from the Superose 6 gel filtration column.

Structurally, K_ATP_ channels are large hetero-octameric complexes of four pore-forming (Kir6.x) and four regulatory sulfonylurea receptor (SURx) subunits [8]. Kir6.x is a member of the inwardly rectifying K^+^ channel family and functions as a tetrameric channel pore permitting transmembrane flux of K^+^ ions [2]. With the exception of vascular smooth muscle, Kir6.2 serves as the pore-forming subunit. SUR belongs to the ABCC subfamily of ATP-binding cassette (ABC) transporter proteins [9]. It endows Kir6.2 with sensitivity to sulfonylurea drugs, K-channel openers and stimulation by Mg-nucleotides. It has 17 transmembrane helices arranged in groups of 5, 6 and 6 (transmembrane domains TMD0, TMD1 and TMD2) (Fig. 1A). The large cytosolic loop between TMD0 and TMD1 interacts both physically and functionally with the N-terminus of Kir6.2 to modulate opening and closing of the pore [10,11]. It also forms part of the sulfonylurea-binding site [12]. The large cytosolic domains following TMD1 and TMD2 contain nucleotide-binding domains (NBD1 and NBD2, respectively) that cooperate in nucleotide binding and hydrolysis [13,14]. It is thought that, like other ABC proteins, the NBDs of SUR associate in a head-to-tail conformation to form two dimeric nucleotide-binding sites (site 1 and site 2) that comprise the Walker A and Walker B motifs of one NBD and the ABC signature sequence of the other.

There are two genes that encode SUR, *ABCC8* (SUR1) and *ABCC9* (SUR2); SUR2 is alternatively spliced to yield several isoforms, the most important being SUR2A and SUR2B [1,2]. The three main isoforms have different sensitivities to drugs and metabolism and a different tissue distribution: SUR1 is found in pancreatic beta-cells and neurones, SUR2A in cardiac and skeletal muscle, and SUR2B in vascular smooth muscle and some neurones.

Most ABC proteins use the energy of ATP hydrolysis to transport a range of substrates across biological membranes. However, SUR is unique in that it serves as an ion channel regulator and has no known transport function; instead, Mg-nucleotide interaction with the NBDs of SUR1 modulates the gating of Kir6.2 and thereby contributes to metabolic sensing by the K_ATP_ channel [15]. Both Kir6.x and SUR subunits participate in the metabolic regulation of channel activity by nucleotides, with binding of ATP to Kir6.2 closing the channel and binding of Mg-nucleotides (MgATP, MgADP) to SUR stimulating channel opening [15–17].

Structural information on the K_ATP_ channel is still limited. The 3D structure of a eukaryotic Kir channel (2.2) has been solved [18], as have those of the cytosolic domains of two related eukaryotic Kir channels [19,20], a bacterial Kir homologue [21] and eukaryotic–prokaryotic chimeric channels [22]. This has enabled construction of a molecular model of Kir6.2 [23]. Information on the complete structure of ABC proteins is also sparse, and in the case of eukaryotic ABC proteins is limited to P-glycoprotein and ABCB10, both of which are not members of the ABCC subfamily and lack TMD0 [24,25] (Dataset coordinates for ABCB10 can be found under accession number 4AYT). A low resolution (18 Å) structure of the complete K_ATP_ channel complex using single-particle analysis of electron microscope (EM) images has also been determined [26]. This suggests that the four SUR subunits may be arranged around a central Kir6.2 tetramer, although this remains to be demonstrated.

Here, we address the location of the constituent subunits within the overall structure using an integrated approach that combines EM and molecular modelling. We use a lipid monolayer based method that exploits the interaction between an HAT-tagged protein and Ni^2+^ attached to the lipid [27–29] to examine single SUR2B particles in the absence of Kir6.2. We show that SUR2B is able to form tetramers independently of Kir6.2, and that the tetrameric structure contains a central space that is large enough to accommodate a tetrameric Kir channel. In our case, affinity capture of the tagged proteins on a lipid monolayer means that the tetramers, each consisting of four HAT-anchored SUR2B subunits, are highly likely to be positioned in the same orientation with respect to the monolayer [30]. This reduces the need for classification to obtain a projection map. Homology modelling was then used to aid interpretation of how the four SUR2B subunits fit into the megadalton membrane protein complex. Our results also provide a first glimpse of how the different domains of SUR may be arranged.

## Results

As previously reported, SUR2B can be expressed in Sf9 insect cells and purified to homogeneity as a fully functional protein that retains ATPase activity [31]. Gel filtration revealed that SUR2B eluted as a single peak corresponding to a single oligomeric species (Fig. 1B). The calculated molecular weight indicated that this species is likely to be a tetramer. No larger aggregates or other protein species were detected. SDS/PAGE and western blotting confirmed that the peak corresponds to SUR2B (Fig. 1C). On SDS/PAGE gels the protein runs as a protomer.

### Single particles

**Fig. 2:**
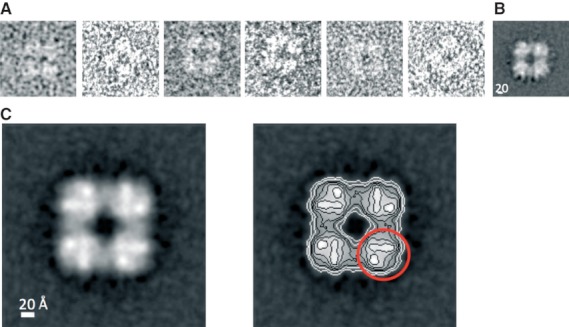
Single SUR2B particles resolved by EM. (A) Typical particles displaying rough four-fold symmetry. Each box is 304 Å across. (B) Non-symmetrized average of 297 particles. (C) Average image and contour map of 297 particles, with C4 symmetry imposed. The red circle indicates a putative SUR2B protomer. The scale bar indicates 20 Å and the box is 304 Å across.

Following purification, SUR2B bearing a C-terminal HAT tag was bound to a fluorinated lipid (phenyl-HF-NTA-Ni) monolayer [32], negatively stained and examined by EM. SUR2B appeared as a homogeneous population of single particles that were square-shaped in cross-section with an unstained central region. Figure 2A shows examples of typical single particles used for analysis. All the particles showed the same orientation, presumably because of unidirectional binding to the lipid monolayer. Figure 2B shows the average of 297 particles, which has a calculated resolution of 21 Å. It is evident that this shows clear four-fold rotational symmetry and thus C4 symmetry was subsequently applied (Fig. 2C).

Surprisingly, only a small amount of HAT-tagged SUR2B protein actually bound to the Ni^2+^ carried by the phenyl-HF-NTA-Ni lipid monolayer. As the monolayer was made with 100% Ni-fluorinated lipids we were expecting a good coverage of the grid with protein but in fact protein coverage was sparse. Increasing the protein concentration did not make any difference. In contrast the fluorinated lipids described previously by Lebeau and colleagues 2001 showed good binding of His-tagged protein. Thus although phenyl-HF-NTA-Ni lipids have been shown to have suitable physicochemical properties (including fluidity at the air–water interface [32]) it appears that they interact less efficiently with the HAT tag. Consequently, although they are suitable for single-particle studies they will not be viable for 2D crystallization of proteins where the amount of protein at the interface needs to be very high.

### Structural details of the SUR2B tetramer

In the absence of nucleotides, the particles form a square-shaped complex ∼ 140 Å in cross-section at its widest point (143 × 140 Å) (Fig. 2B,C). There is a clear lack of density in the centre of the structure, which is roughly quatrefoil shaped and ∼ 45–50 Å across. The dimensions and topology suggest that each particle corresponds to a tetramer of SUR2B subunits that surround a central ‘hole’. We propose that this cavity is where the Kir6.2 tetramer fits. Regions of higher density are seen at each of the four corners of the complex (Fig. 2C, circled), which are roughly circular and presumably correspond to individual SUR2B protomers. These are linked by a narrower region of lesser density that measures ∼ 31 × 15 Å. Within each of the four protomers, some further structure can be discerned: in particular, there appear to be two regions of higher density located towards the outer part of the protomer and a single region of higher density that is found towards the inner part of the structure.

### Fitting of ABC X-ray structures into the maps

**Fig. 3:**
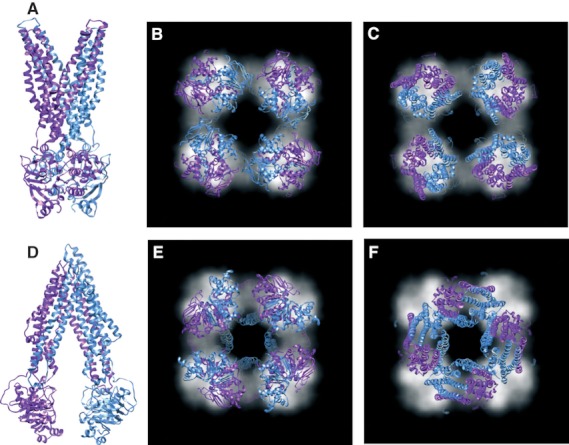
Fits of ABC X-ray structures into the EM maps. (A) Structure of the Sav1866 homodimer (one monomer is coloured blue and the other purple). (B, C) X-ray structure of Sav1866 homodimer viewed (B) from the intracellular side (hence NBDs are to the front) and (C) from the extracellular side (i.e. TMDs face the front). The 2D EM projection map of SUR2B was positioned at the NBD–TMD interface, in the fitted *x*, *y* orientation, in order to visualize the overlay with the NBDs (B) or the TMDs (C) of the 3D Sav1866 structure (see Fig. 4). The arrow indicates the difference in viewpoint between (B) and (C, D) Structure of P-glycoprotein (TMD1–NBD1 is coloured blue, TMD2–NBD2 purple). (E, F) X-ray structure of P-glycoprotein viewed (E) from the intracellular side (hence NBDs are to the front) and (F) from the extracellular side (i.e. TMDs face the front). The 2D EM projection map of SUR2B was positioned at the NBD–TMD interface, in the fitted *x*, *y* orientation, in order to visualize the overlay with the NBDs (E) or the TMDs (F) of the 3D P-glycoprotein structure (see Fig. 4). The arrow indicates the difference in viewpoint between (E) and (F).

No X-ray structure of SUR exists. However, the structure of the TMD1–NBD1–TMD2–ND2 core of SUR is likely to be similar to that of canonical ABC exporters such as P-glycoprotein and Sav1866, the structures of which are available in both nucleotide-free (PgP) and nucleotide-bound forms (Sav1866) [24,33]. Therefore, we fitted the crystal structures of both Sav1866 (NBDs closed) and PgP (NBDs open) into the EM maps to determine which conformation to use as a template for the SUR2B model (Fig. 3). This was done by first converting the 3D structure of Sav1866 (or PgP) into a 2D projection map and then fitting this to the 2D EM map of the SUR2B tetramer. This enabled the best fit of the *x* and *y* orientations of the 3D structures to the SUR2B projection map to be obtained. For display purposes, the figures show the 2D EM map of the SUR2B tetramer superpositioned on the fitted 3D structures of Sav1866 (Fig. 3A–C) or PgP (Fig. 3D–F).

The fits of the Sav1866 structure are shown in Fig. 3A–C. The handedness of the 2D EM maps is unknown, but fitting into mirror images of the EM map gave the same orientation of Sav1866. EM maps accommodate four copies of the Sav1866 structure well, with the helical subdomain of one NBD positioned closest to the central hole (Fig. 3B,C).

Fits of the PgP structure indicate that the EM maps accommodate it less well than the Sav1866 structure: the NBDs either stick out of the EM map or clash with the NBDs of neighbouring protomers (Fig. 3E,F). This suggests that the TMD1–NBD1–TMD2–NBD2 core of SUR2B adopts a conformation that is rather similar to the nucleotide-bound Sav1866 structure, in which the NBDs and the ‘cytosolic necks’ of the TMDs are packed tightly together.

### Homology model fitting

**Fig. 4:**
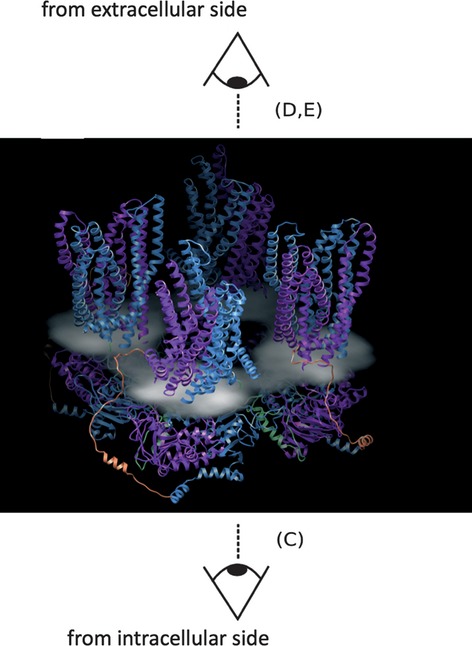
Homology model of SUR2B. TMD1 and NBD1 are coloured blue, TMD2 and NBD2 are coloured purple. The 2D EM projection map was positioned at the NBD–TMD interface of the 3D model. The ‘eyes’ indicate the direction from which the models are viewed in Figs 3 and 5.

**Fig. 5:**
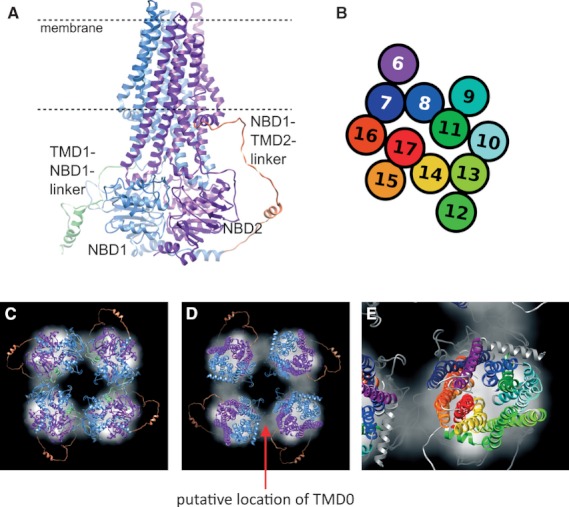
Homology model of SUR2B, lacking TMD0 and CL3. (A) Homology model of SUR2B. TMD1 and NBD1 are coloured blue, TMD2 and NBD2 are coloured purple. Two long loops not included in the structural template are coloured green (TMD1–NBD1 linker) and red (NBD1–TMD2 linker). The approximate location of the membrane is indicated. (B) Schematic topology of TM helix packing looking from the extracellular side. Helices are coloured in a rainbow scheme, as indicated. (C–E) Fits of the SUR2B homology model into the EM projection map. TMD1 and NBD1 are coloured blue, TMD2–NBD2 is purple, the TMD1–NBD1 linker is light green and the NBD1–TMD2 linker is light red. (C–E) SUR2B model viewed (C) from the intracellular side (hence NBDs are to the front) and (D) from the extracellular side (i.e. TMDs face the front). As in Fig. 4, the EM projection map was positioned at the NBD–TMD interface, in the fitted *x*, *y* orientation, to show the overlap of individual domains (and is always viewed from the same orientation). The arrow indicates the difference in viewpoint between (C) and (D). (E) Close-up view of the TM helices [colouring as in (B)].

We next constructed a homology model of SUR2B using the Sav1866 structure as a template. No high-resolution structural information exists on which to model the TMD0 and CL3 regions of SUR, and these were therefore omitted. Figure 4 shows how the SUR2B model was superpositioned on the SUR2B EM projection map. Figure 5 shows the model of a single SUR2B subunit (A,B) and the fit of four such subunits into the EM map (C–E).

When fitting the homology model we cannot distinguish a fitted orientation from its dimer-related counterpart (i.e. the same fit rotated by 180°). In other words, the fitting statistics cannot tell which NBD and which TMD sit closer to the centre of the tetramer. We selected NBD1 as the NBD whose helical subdomain sits closer to the central hole, but did not bias the fitting of SUR2B homology models in any other way.

The best-fitted SUR2B homology models adopt orientations very similar to those seen for fitted Sav1866 structures (Fig. 5C–E). The helical subdomain of NBD1 reaches into the central hole. Nucleotide-binding site 2, which is formed by the catalytic domain of NBD2 and the helical subdomain of NBD1, is thus close to the channel interior. By contrast, the distance between opposing SUR2B subunits at the level of the membrane is larger. While we cannot reliably model the structures of the major loops of SUR2B, their location relative to the protein core should be correct in our homology models. Thus, the fits in Fig. 5C–E show that the TMD1–NBD1 linker is located at the putative SUR–Kir6.x interface. This linker contains the endoplasmic reticulum retention motif, which has to be masked in the octameric complex to allow trafficking [34], so a position at the inter-subunit interface is sensible. The other large unmodelled loop, the NBD1–TMD2 linker, is predicted to be on the outside of the SUR2B tetramer.

### Location of TMD0

The fits of the Sav1866 and SUR2B model structures leave additional unoccupied EM density between the fitted structures. This is likely to be occupied by the TMD0 and CL3 regions of SUR2B. In this relative orientation, TMD0 would interact with TMD1 of one protomer and TMD2 of another protomer. TMD0 would also form a large interaction surface with TM regions of Kir6.x.

### Location of Kir62 tetramer

**Fig. 6:**
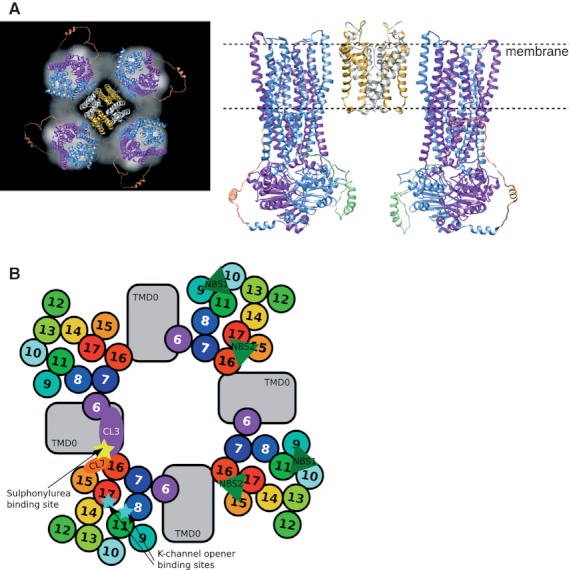
Schematic illustration of the proposed TM helix packing in the SUR tetramer. (A) Homology model of SUR2B and the TMDs of the crystal structure of Kir2.2 fitted into the EM map and viewed from the extracellular side of the membrane (left) or shown parallel to the plane of the membrane (right). TMD1 and NBD1 are coloured blue, TMD2 and NBD2 are coloured purple. Two long loops not included in the structural template are coloured green (TMD1–NBD1 linker) and red (NBD1–TMD2 linker). Two protomers of Kir2.2 are coloured gold and two are silver. (B) Schematic topology of TM helix packing looking from the extracellular side. Helices are coloured in a rainbow scheme, as indicated. Positions of the nucleotide-binding sites relative to the TM helices are indicated by green triangles. Positions of the presumed drug-binding sites are shown by yellow and cyan stars.

The crystal structure of the Kir2.2 tetramer fits easily into the central hole of the SUR2B tetramer at the level of TMDs (Fig. 6). The orientation of the Kir tetramer was selected so as to minimize clashes between its transmembrane helices and those of SUR. Models of Kir6.2 based on the structure of Kir2.2 fit equally well. At the levels of the cytosolic domains significant overlap between Kir2.2 and SUR occurs which cannot be remedied by rotation about the central axis. However, we note that translation of the Kir cytosolic domain towards the membrane, as has been proposed to occur during channel gating [22], would remove many such clashes (see Fig. S2).

### Comparison to an EM map of the entire K_ATP_ complex

**Fig. 7:**
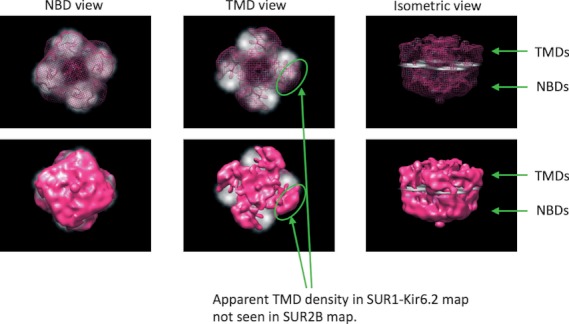
Comparison of the SUR2B tetramer map to an EM map of the entire K_ATP_ complex. EM densities of the NBDs of SUR2B (white) and the K_ATP_ complex (pink). The density of the Kir6.2–SUR1 complex does not completely overlie the 2D projection of SUR2B alone, as marked by the green ovals.

A comparison of the SUR2B tetramer with an earlier single-particle 3D reconstruction of the complete octameric Kir6.2–SUR1 complex [26] is shown in Fig. 7. The NBD regions of the earlier reconstruction closely match the outline of the SUR2B projection map presented here. Furthermore, the square-shaped density in the centre of the K_ATP_ channel complex, which was assumed to be the Kir6.2 tetramer [26], also overlies well with the central square-shaped hole in the SUR2B map.

However, at the level of the TMDs, a small part of the Kir6.2–SUR1 map is not accounted for in our map of the SUR2B tetramer. As Fig. 7 shows, the SUR2B projection map appears to be rotated and/or radially displaced relative to the 3D reconstruction in this region. There are several possible explanations for this difference. First, the presence of Kir6.2 may push the TMDs further apart: as indicated above, our modelling studies indicate there is insufficient room to fit the cytosolic domains of the Kir2.2 tetramer within the central cavity of the SUR2B tetramer without a clash with SUR. This clash is predicted to be even larger in the open state, when it is believed that the cytosolic domains of Kir move upwards and rotate [35,36]. Thus the TMDs may need to move to accommodate Kir6.2. Alternative explanations include (i) the fact that different detergents were used (Fos16 here versus DDM in the Kir6.2/SUR1 channel [26]) which potentially may add an outer shell of different size; (ii) it is possible that the two proteins are not in the same state, because the SUR2B tetramer, but not the Kir6.2–SUR1 complex, was studied in the presence of glibenclamide.

## Discussion

### Tetramerization of SUR2B

Our results clearly demonstrate that SUR2B can form tetramers in the absence of Kir6.2. This was evident both from the elution profile of purified SUR2B and from the appearance of the particles observed by EM. The tetrameric nature of SUR2B is consistent with a previous study in which the isolated NBDs of SUR1 were found to elute as a complex with a size equivalent to eight to nine individual subunits on size-exclusion chromatography and to form a ring-like structure when viewed by EM 2007. Our studies suggest that tetramerization is intrinsic to SUR and does not require the presence of Kir6.2. As the NBDs of SUR1 can associate in the absence of the TMDs 2007, it is clear that tetramerization of SUR involves the NBDs, but this does not of course exclude the possibility that the TMDs are also involved. Our data also confirm that Kir6.2 sits within an outer ring of SUR, which will almost fully embrace the tetrameric pore.

The outer diameter of the SUR2B tetramer was 143 Å. This compares favourably with the outer diameter of the ring-like structure of the isolated NBDs of SUR1 (120–140 Å) 2007 but is slightly smaller than that of the purified octameric Kir6.2–SUR1 complex (180 Å) [26]. This may reflect differences in the experimental approach (e.g. cryo-negative staining for Kir6.2–SUR1 versus negative staining for the isolated NBDs and in this study) or the presence of the Kir6.2 tetramer within the purified complex.

The SUR2B tetramer is roughly square-shaped, with a circular central cavity that has an inner maximum diameter of ∼ 50 Å. This is within the range (40–75 Å) of that reported for the isolated SUR1–NBD ring 2007. The widest diameter of the cytoplasmic domain of the Kir channels Kir2.1, Kir3.1 and Kir3.2 was ∼ 80 Å in the crystal structure. Thus the central cavity may increase slightly to accommodate the cytosolic domains of the Kir6.2 tetramer [26]; this might explain why the overall diameter of the Kir6.2–SUR1 complex was somewhat larger than that of the SUR2B tetramer we report here.

Interestingly, the unrelated ABC protein ABCG2 also forms a tetrameric complex [38]. The ABCG2 gene encodes a ‘half-transporter’ consisting of a single TMD and a single NBD that, unusually, is located N-terminal to the TMD. It assembles as a homodimer and then as a tetramer of homodimers. The ABCG2 tetramers are of a similar size to those of SUR2B, being square-shaped, ∼ 170 Å in width and with a central hole of ∼ 60 Å [38]. Another single-particle analysis of ABCG2 showed it bound to a lipid monolayer as a tetramer of protomers, resulting in ring-shaped structures with an overall size of about 120 Å [39].

### Fits of the model to the EM map

When fitting the SUR2B model into the EM density, we had to decide manually between two possible orientations that are related by a rotation by 180º. We chose an orientation of SUR2B in which the helical subdomain of NBD1 is closest to the interior of the complex. The location of the fitted SUR2B model within the complex is in agreement with much previous work. For example, studies have shown that NBD1 of SUR1 is crucial for assembly of SUR1 [40] and that NBD1 associates as a tetramer [41] or octamer 2007. This predicts that the NBD1s of adjacent SUR1 will interact. Significant interactions between NBD1 and NBD2 have also been demonstrated in both biochemical and functional studies. Thus co-expression of TMD2–NBD2 with NBD1–GFP, but not of TMD2 and NBD1–GFP, resulted in the membrane localization of NBD1 [41]. Furthermore, mutations in NBD2 impair high-affinity ATP binding to NBD1 [42]. Close interactions of NBD1–NBD2 are also seen in our homology model of SUR2B (a direct consequence of the Sav1866 template structure).

Our model of the tetrameric SUR2B complex suggests that neither TMD1 nor TMD2 of one subunit interact with the equivalent domains in the adjacent subunit. Nevertheless, we cannot exclude the possibility that interactions between the TMDs of SUR2B of adjacent subunits exist. It is quite possible that if TMD0 sits between two subunits (see below) it will interact not only with TMD1 of its own subunit but with TMD2 of the adjacent subunit.

### Location of TMD0

Unlike most ABC proteins, SUR contains an additional set of five transmembrane segments (TMD0) N-terminal to TMD1, as do MRP1 and some other proteins of the ABCC subfamily [43]. The ABC transporter Tap1/2 features N-terminal additions of four TM segments. Similar to TMD0 of SUR, the N-terminal extension in Tap1/2 is required for interaction with other proteins [44]. Our EM map and modelling offer some suggestion of where TMD0 might be located in the SUR2B tetramer. There is a region of lower density between the adjacent SUR2B subunits that is not filled by TMD1 or TMD2 of the homology model. It seems plausible that TMD0 sits here, between the two SUR subunits. A similar suggestion was made for SUR1 by [26], based on a low resolution EM structure of the entire K_ATP_ complex.

### Putative location of drug-binding sites

There is considerable evidence that the glibenclamide-binding site involves residues in the cytosolic loops 3 (CL3) and 8 (CL8, sometimes referred to as CL7), which lie between TMD0 and TMD1, and TM15 and TM16 respectively [12,45,46]. Specifically, mutation of serine 1237 of SUR1 to tyrosine abolishes the high-affinity block of channel activity by tolbutamide [45] whereas the reverse mutation in SUR2 confers tolbutamide sensitivity [47]. The S1237Y mutation also reduces [^3^H]-glibenclamide binding to SUR1 and renders channel inhibition by glibenclamide readily reversible [45]. In addition, mutation of tyrosine 230 in CL3 to alanine abolishes I^125^-glibenclamide binding [46], implicating CL3 as well as CL8 in sulfonylurea binding. The glibenclamide-binding site must also lie fairly close to Kir6.2 because Kir6.2 can be photoaffinity labelled with azido-glibenclamide [8].

Our model places transmembranes 6 and 16 on the same face of SUR, facing towards Kir6.2. Based on this location, we speculate that CL8 of one SUR2B is likely to be located close to CL3 of the adjacent SUR2B, as well as to Kir6.2. This would put the glibenclamide-binding site at the interface between adjacent SUR subunits. Interestingly, binding sites are generally found at the interface between protein subunits or domains. This is true for the acetylcholine-binding site of the acetylcholine-binding protein [48], the ATP-binding site of Kir6.2 [23] and even the nucleotide-binding domains of SUR2B itself. The position at the interface between two subunits may help account for the fact that glibenclamide appears to be able to act as a chaperone, escorting mutant SURs to the plasma membrane [49,50].

In conclusion, our results demonstrate that SUR2B forms tetramers in isolation. This indicates that SUR2B is not simply passively assembled on a Kir6.2 template but that it has intrinsic tetramerization capability, in contrast to most other ABC proteins.

## Materials and methods

### Expression and purification of SUR2B

An HAT tag (Clontech) was added to the C terminus of rat SUR2B, and the gene was expressed in insect cells (Sf9) using a baculovirus expression system (Invitrogen), essentially as described for SUR1 2007. Protein expression was verified by [^3^H]-glibenclamide binding to infected Sf9 cells 48 h after infection [26].

Cells were lysed under high pressure and membranes were purified by centrifugation. Membranes were solubilized for 2 h in a buffer containing 50 mm Tris (pH 8.0), 150 mm NaCl, 300 mm sucrose, 5 mm MgCl_2_, 2 mm TCEP and 1% w/v Fos-16 (Analytical grade, Affymetrix). All buffers in subsequent steps contained 10 μm glibenclamide, 2.5% sucrose (instead of 300 mm) and 0.01% w/v Fos-16 (except for gel filtration buffer which contained 0.001% w/v Fos-16).

Protein was purified by Co^2+^ affinity chromatography followed by a gel filtration step using Superose-6 column (GE Healthcare). Peak fractions were pooled and concentrated to 1 mg·mL^−1^. Purified protein averaged 100 μg·L^−1^ Sf9 culture. Proteins were analysed on 4%–12% gradient Bistris SDS/PAGE gels and visualized by Coomassie staining (Invitrogen).

### Monolayer formation and protein capture

A Teflon® block containing 70 μL wells, each with a small, angled, side tube, was used for the experiments. Wells were initially filled with 70 μL of buffer A containing 50 mm Tris (pH 8.0), 150 mm NaCl, 10 mm TCEP, 10 μm glibenclamide, 2.5% sucrose and 0.1% sodium azide. Using a Hamilton microsyringe, 1 μL of 0.5 mm fluorinated lipid (phenyl-HF-NTA-Ni) [32] in chloroform/hexane (1:1 v/v) was added to the top of the solution. The block was placed in a humidified container and incubated at 20 ºC for 24 h.

A stock solution of DOPC:DHPC (5 mg·mL^−1^ 1,2-dioleoyl-sn-glycero-3-phosphocholine plus 10 mg·mL^−1^ of 2-diheptanoyl-sn-glycero-3-phosphocholine, from Avanti Lipids) in deionized water was prepared and mixed with 1 mg·mL^−1^ protein in buffer A plus 0.001% Fos-16 at a ratio of 0.8 lipid to protein. This was added to the well via the side tube without disturbing the lipid monolayer so the final concentration of proteins in the well was 70–80 μg·mL^−1^. After 24 h equilibration, bio-beads were added via the side tube (to facilitate the removal of the detergent from the lipid bilayer) and incubated for a further 24 h. The fluorinated lipid monolayer (along with the immobilized HAT-tagged protein in a DOPC bilayer) was then transferred to hydrophobic carbon coated grids using the Langmuir–Schaefer deposition method, stained with 2% w/v uranyl acetate and examined with a Philips CM120 electron microscope.

### Electron microscopy and image processing

Images were recorded on Kodak SO 163 film using a Philips CM120 electron microscope operating at 120 kV under low dose conditions with a nominal magnification of 45 000 × (actual magnification 41 000 ×). Images were digitized with a step size of 12.5 μm on a Nikon Super Coolscan 9000. The pixel size was 3.04 Å. Particles were picked by hand, masked and aligned with a reference-free method [51]. The web and spider software package [52] was used for all image processing. All particles (297) imaged were used. Resolution was assessed from the 0.5 Fourier-shell correlation cut-off [52,53].

### Modelling of SUR2B

A homology model of human SUR2B was built using modeller 9v5 [54] and the ADP-bound crystal structure of Sav1866 (PDB ID 2HYD) as template. A multiple sequence alignment of SUR2B, SUR1 and Sav1866 was created with clustalx 2.1 [53] by aligning the half-transporters of SUR (TMD1–NBD1 and TMD2–NBD2) individually with Sav1866. The sequence alignments were combined to yield a complete alignment of SUR2B (residues 281–1549) with homodimeric Sav1866 (1156 residues). This alignment is shown in Fig. S1. The sequence identity is 21% overall, with higher identity in the NBDs (30–35%) and lower identity in the TMDs (10–14%) (Fig. S1). Loop insertions of SUR2B that are not present in Sav1866 were constructed using the loop modelling feature of modeller 9.

### Fitting of structures and models into the projection map

Crystal structures of Sav1866 (PDB ID 2HYD), P-glycoprotein (PDB ID 3G5U) and a homology model of SUR2B (based on the Sav1866 structure) were fitted to the EM maps using spider [52]. In this process, the 3D structures were first converted into 3D density maps using a Gaussian filter and centred on the origin. Next, the density was rotated in steps of 1° around the *z*-axis and projected along the *z*-axis to yield a 2D projection map of the crystal structure or model. For each rotation angle step, a cross-correlation and peak search was used to identify the best fit of this 2D projection into the SUR2B EM map, as described by Kuo *et al*. 2005. In order to fit three other protomers into the same EM map, the fitting was repeated at fixed rotation angles of +90°, +180° and +270° relative to the rotation angle of the optimal fit. The resulting rotations and shifts were also applied to the PDB file itself, resulting in positions and orientations that correspond to the best fits. This enabled the best fit of the *x* and *y* orientations of the structures/model to the SUR2B projection map to be obtained.

For display purposes, in the figures the 2D SUR2B EM map is displayed in conjunction with the fitted 3D structure (or model). The EM map was positioned at the NBD–TMD interface and the 3D structure was then viewed from either the intracellular or extracellular side (Figs 4 and 5). The orientation of the projection map was not changed. The EM map and fitted structures were visualized in ucsf chimera [56].

The TM regions (residues 70–183) of the Kir2.2 crystal structure (PDB ID 3JYC) were tetramerized using crystallographic symmetry transformations. The resulting structure was positioned manually within the SUR2B tetramer using chimera. Rotation of the channel around its central axis yielded a fit with no atomic clashes with the TMDs of the SUR model.

In order to compare the 2D EM map of purified SUR2B with an earlier 3D single-particle reconstruction of the complex octameric Kir6.2–SUR1 complex [26], the two maps were superpositioned such that they shared the same symmetry axis and rotated relative to each other around this axis so that the 2D map best matched the projection of the 3D map. ucsf chimera was used for the manual overlay and visualization [56].

## Abbreviations

ABC: ATP-binding cassette
K_ATP_: ATP-sensitive potassium
Kir6.x: inwardly rectifying potassium channel (pore-forming subunit)
NBD: nucleotide-binding domain
SUR: sulfonylurea receptor
TMD: transmembrane domain

## Supporting information

**Fig. S1.** Sequence alignment of SUR2B and Sav1866.

**Fig. S2.** Homology model of the KATP channel complex SUR2B and Kir22.
