# Supplementary material for: Tetrameric structure of SUR2B revealed by electron microscopy of oriented single particles
Source: FEBS J. 2013 Jan 27;280(4):1051–63. doi: 10.1111/febs.12097 (PMC3599479; doi:10.1111/febs.12097)
Supplement: Supplementary file 1 [file febs0280-1051-SD1.zip › febs12097-sup-0001-FigS1-S2.pdf]

the FEBS Journal

www.febsjournal.org

Tetrameric structure of SUR2B revealed by electron microscopy of oriented single particles

Constantina Fotinou, Jussi Aittoniemi, Heidi de Wet, Ange Polidori, Bernard Pucci, Mark S. P. Sansom, Catherine Vénien-Bryan and Frances M. Ashcroft

DOI: 10.1111/febs.12097

Supplementary Figure 1

```
SUR2B_res_281_1549   RTPSIWLAMYRAFGRPILLSSTFRYLADLLGFAGPLCISGIVQRVNETQ-NGTNNTTGIS--ETLSSKEFLENAYVLAVLLFLALILQRTFLQASY-YVTIETGINLRGALLAMIYNKILRLSTSNLSMGEMTLGQINNLVAIETNQLMW
Sav1866_two_monomers ---------MIKRYLQFVKPYKYRIFATIIVGIIKFGIPMLIPLLIKYAIDGVI------NNHALTTDEKVHHLTIAIGIALFIFVIVRPPIEFIRQYLAQWTSNKILYDIRKKLYNHLQALSAR--FYANNQVGQVISRVINDVEQTKD

SUR2B_res_281_1549   FLFL-CPNLWAMPVQIIMGVILLYNLLGSSALVGAAVIVLLAPIQYFIATKLAEAQKSTLDYSTERLKKTNEILKGIKLLKLYAWEHIFCKSVEETRMKELSSLKTFALYTSLSIFMNAAIPIAAVLATFVTHA-YASGNNLKPAEAFAS
Sav1866_two_monomers FILTGLMNIWLDCITIIIALSIMFFLDVKLTLAALFIFPFYILTVYVFFGRLRKLTRERSQALAEVQGFLHERVQGISVVKSFAIEDNEAKNFDKKNTNFLTRALKHTRWNAYSFAAINTVTDIGPIIVIGVGAYLAISGSITVGTLAAF

SUR2B_res_281_1549   LSLFHILVTPLFLLSTVVRFAVKAIISVQKLNEFLLSDEIGDDSWRTGESSLPFESCKKHTGVQPKTINRKQPGRYHLDSYEQSTRRLRPAETEDIAIKVTNGYFSWG-SGLATLSNIDIRIPTGQLTMIVGQVGCGKSSLLLAILGEMQ
Sav1866_two_monomers VGYLELLFGPLRRLVASFTTLTQSFASMDRVFQLI-----------------------------------------------------------KQGRIDIDHVSFQYNDNEAPILKDINLSIEKGETVAFVGMSGGGKSTLINLIPRFYD
                                                   <------------------- TMD1-NBD1 linker ------------------->

SUR2B_res_281_1549   TLEGKVHWSNVNESEPSFEATRS-----RNRYSVAYAAQKPWLLNATVEENITFGSPFNKQRYKAVTDACSLQPDIDL---LPFGDQTEIGERGINLSGGQRQRICVARALYQNTNIVFLDDPFSALDIHLSDHLMQEGILKFLQDDKRT
Sav1866_two_monomers VTSGQILIDG---------HNIKDFLTGSLRNQIGLVQQDNILFSDTVKENILLGRPTATD--EEVVEAAKMANAHDFIMNLPQGYDTEVGERGVKLSGGQKQRLSIARIFLNNPPILILDEATSALDLE-SESIIQEALDVLSKD--RT

SUR2B_res_281_1549   LVLVTHKLQYLTHADWIIAMKDGSVLREGTLKDIQTKDVELYEHWKTLMNRQDQELEKDMEADQTTLERKTLRRAMYSREAKAQMEDEDEEEEEEEEDEDDNMSTVMRLRTKMPWKT-CWRYLTSGGFFLLILMIFSKLLKHSVIVAIDYW
Sav1866_two_monomers TLIVAHRLSTITHADKIVVIENGHIVETGTHRELIAKQGA-YEHLYSIQNL------------------------------------------------------------------MIKRYLQ--FVKPYKYRIFATIIVGIIKFGIPML
                                                               <------------------- NBD1-TMD2 linker ------------------->

SUR2B_res_281_1549   LATWTS----------EYSINNTGKADQTYYVAGFSILCGAGIFLCLVTSLTVEWMGLTAAKNLHHNLLNKIILGPIRFFDTTPLGLILNRFSADTNIIDQHIPPTLESLTRSTLLCLSAIGMISYATPVFLVALLPLGVAFYFI--QKY
Sav1866_two_monomers IPLLIKYAIDGVINNHALTTDEKVHHLTIAIGIALFIFVIVRPPIEFIRQYLAQWTSNKILYDIRKKLYNHLQALSARFYANNQVGQVISRVINDVEQTKDFILTGL--MNIWLDCITIIIALSIMFFLDVKLTLAALFIFPFYILTVYV

SUR2B_res_281_1549   FRVASKDLQELDDSTQLPLLCHFSETAEGLTTIRAFRHETRFKQRMLELTDTNNIAYLFLSAANRWLEVRTDYLGACIVLTASIASISGSSN----SGLVGLGLLYALTITNYLNWVVRNLADLEVQMGAVKKVNSFLTMESENYEGTMD
Sav1866_two_monomers FFGRLRKLTRERSQALAEVQGFLHERVQGISVVKSFAIEDNEAKNFDKKNTNFLTRALKHTRWNAYSFAAINTVTDIGPIIVIGVGAYLAISGSITVGTLAAFVGYLELLFGPLRRLVASFTTLTQSFASMDRVFQLIDEDYDIKNGVGA

SUR2B_res_281_1549   PSQVPEHWPQEGEIKIHDLCVRYENNLKPVLKHVKAYIKPGQKVGICGRTGSGKSSLSLAFFRMVDIFDGKIVIDGIDISKLPLHTLRSRLSIILQDPILFSGSIRFNLDPEC-KCTDDRLWEALEIAQLKNMVKSLPGGLDAVVTEGGE
Sav1866_two_monomers QPIEIK----QGRIDIDHVSFQYNDNEAPILKDINLSIEKGETVAFVGMSGGGKSTLINLIPRFYDVTSGQILIDGHNIKDFLTGSLRNQIGLVQQDNILFSDTVKENILLGRPTATDEEVVEAAKMANAHDFIMNLPQGYDTEVGERGV

SUR2B_res_281_1549   NFSVGQRQLFCLARAFVRKSSILIMDEATASIDMATENILQKVVMTAFADRTVVTIAHRVHTILTADLVIVMKRGNILEYDTPESLLAQENGVFASFVRADM
Sav1866_two_monomers KLSGGQKQRLSIARIFLNNPPILILDEATSALDLESESIIQEALDVLSKDRTTLIVAHRLSTITHADKIVVIENGHIVETGTHRELIAKQGAYEHLYSIQNL
```

Supplementary Figure 1: Sequence alignment of SUR2B and Sav1866

Sequence alignment of human SUR2B (residues 281-1549) and two copies of Sav1866, as was used as a basis for homology modelling. Asterixes indicate pairs of identical residues, colons and dots indicate closely and loosely similar residues.

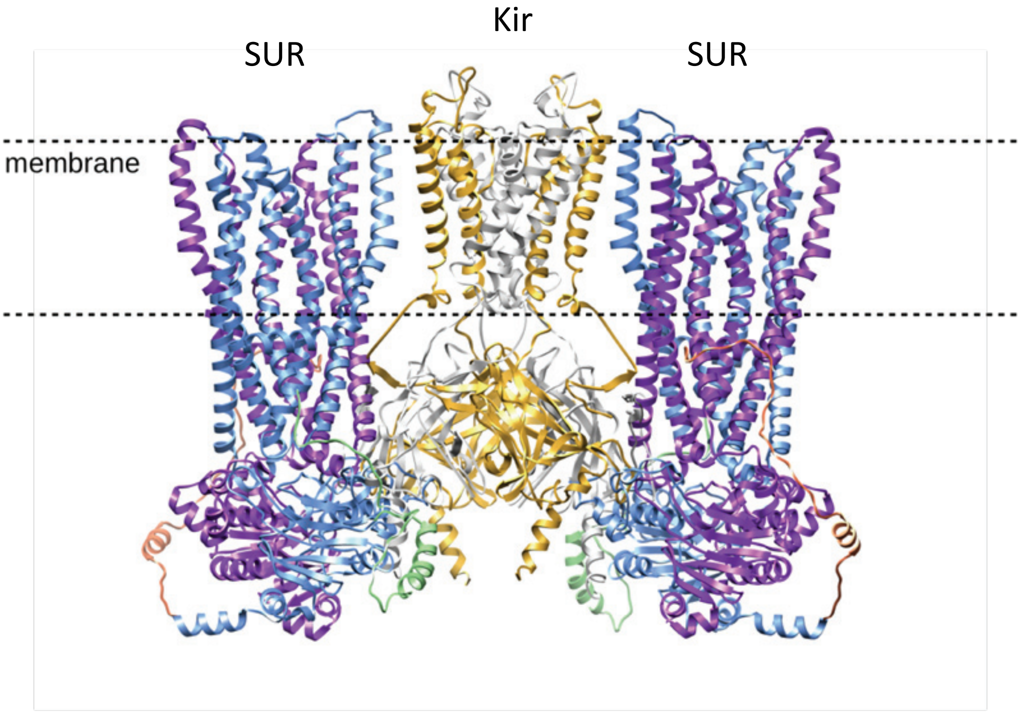

Supplementary Figure 2: Homology model of the K_{ATP} channel complex SUR2B and Kir2.2

A homology model of SUR2B and a crystal structure of Kir2.2 are shown parallel to the plane of the membrane. For SUR2B, TMD1 and NBD1 are coloured blue, TMD2 and NBD2 are coloured purple. Two long loops not included in the structural template are coloured green (TMD1-NBD1 linker) and red (NBD1- TMD2 linker). For Kir2.2, two protomers are coloured gold and two are coloured silver. It is evident that although the transmembrane domains fit well there is some clash within the intracellular domains.
